# Backup of Renewable Energy for an Electrical Island: Case Study of Israeli Electricity System—Current Status

**DOI:** 10.1155/2014/609687

**Published:** 2014-01-29

**Authors:** A. Fakhouri, A. Kuperman

**Affiliations:** Department of Electrical Engineering and Electronics, Ariel University, 40700 Ariel, Israel

## Abstract

The paper focuses on the quantitative analysis of Israeli Government's targets of 10% renewable energy penetration by 2020 and determining the desired methodology (models) for assessing the effects on the electricity market, addressing the fact that Israel is an electricity island. The main objective is to determine the influence of achieving the Government's goals for renewable energy penetration on the need for backup in the Israeli electricity system. This work presents the current situation of the Israeli electricity market and the study to be taken in order to assess the undesirable effects resulting from the intermittency of electricity generated by wind and solar power stations as well as presents some solutions to mitigating these phenomena. Future work will focus on a quantitative analysis of model runs and determine the amounts of backup required relative to the amount of installed capacity from renewable resources.

## 1. Introduction

The growing use of renewable energy requires additional techniques and analyses of its influences on the economy and the reliability of the electrical system in order to provide the system operator the tools to compensate for the intermittency of renewable sources in real time, considering the actual reaction times of the fossil and pumped storage units and their availabilities to back up renewables [1–3]. The challenge of combining energy storage units with the electrical network, aiming towards mechanisms for smart consumption and encouraging flexible generation, is becoming feasible in light of recent developments in communications and smart grid. While it is clear to all that, in parallel with developing storage technologies, it is necessary to develop smart grids that will be able to provide a solution and tools to the system operator to overcome the undesirable phenomena stemming from the intermittency of renewable energy sources. Nevertheless, the value of energy storage is captured best with additional renewable energy generation, thus reducing the use of conventional generation. Valuing the function of storage with renewable sources requires continuous analysis, improved data logging, and developing new techniques in order to assess the activity of smarter and more dynamic networks in the future.

The Government of Israel has recently imposed the following target for renewable energy penetration: 10% of Israel's energy needs should be provided by renewable sources by 2020. The target is expected to be achieved with total installed generation capacity of 2,760 MW. Given the current characteristics of the Israeli market, as will be noted later, this target should be reached using primarily solar and wind facilities. Achieving this target will probably have implications on the need for backup power in the Israeli electricity market, which has the unique characteristics of an “electricity island,” that is, is isolated with no interconnections with neighboring countries. This level of generation in a market whose current installed capacity is 12,769 MW and expected to reach 15,137 MW by 2020 makes renewable energy a significant component of total installed capacity. This significance will only increase when the nondispatchability of wind-based and solar-based renewables is taken into account [4–6].

The main objective of this paper is to determine the influence of achieving the Government's goals for renewable energy penetration on the need for backup in the Israeli electricity system. This work presents the current situation and the expected undesirable effects resulting from the intermittency of electricity generated by wind and solar power stations as well as solutions to mitigating these phenomena. In fossil-based power stations, it is possible to store fuels on the site and supply fuel to the stations continuously. Alternatively, it is possible to decrease or increase load in accordance with the requirements of the system operator. In professional terms, these stations are called “dispatchable.” On the contrary, wind and solar stations rely on wind and irradiation intensity that are not controllable by the power station owner and the system operator; that is, they are “nondispatchable.” This operational deficiency is expressed in the assumption regarding the need for other resources—fossil units, pumped storage, demand response, electronic and chemical storage technologies, and so forth in order to maintain required system reliability; that is, the assumption is that, without these backup resources, replacing fossil plants with renewable plants, at least at a certain replacement level, is likely to cause system reliability and survivability to fall below set targets. The conflict between reliability and survivability targets for the electricity system and targets for renewable energy integration into the electricity market poses challenges to long-term planning and policy making for the Israeli electricity market. This is doubly important given the fact that the Israeli electricity network is an “electricity island” [7, 8].

The paper investigates the backup needs and their characteristics, assuming that the Government's goal of integration renewable energy source at a level of 10% by 2010 is achieved. In order to calculate the backup capacity required for renewables, SAS software will be used to calculate the static coincidence factor for instability from all future planned renewable technologies together and for presenting various solutions by constructing a model to forecast the real-time output of renewable power stations including examining various backup possibilities in order to minimize the need for fossil resources for system backup. The rest of the paper is organized as follows.  Section 2 presents the current electricity market in Israel.  Section 3 revises the Government's targets for integrating renewable energy.  Section 4 reveals the technical issues resulting from the intermittency of these technologies and presents possible solutions.  Section 5 demonstrates various methodologies and models for analyzing the undesirable phenomena associated with renewable energy penetration forecasting the output of renewable power stations, encouraging flexible power stations, and developing storage technologies.  Section 6 concludes the paper.

## 2. Presentation of the Current Israeli Electricity Market, Focusing on the Generation Sector

### 2.1. Generation

The vast majority of electricity is produced by the Israel Electric Corporation (IEC). IEC employs a variety of stations and various fuel mixes in order to respond to Israel's electricity demand. Its total generating capacity (maximum capacity that the company is capable of providing at a given time) is 12,769 MW as of June 1, 2012. The Company's fleet of generating stations is as follows:

- 22 steam units (based on natural gas, oil, or coal) comprise the majority of the power stations along the Mediterranean coast: they use water for cooling and are therefore located along the seacoast; alongside most of the steam units, there are gas turbines and combined-cycle gas turbines with lower capacities as well;
- 31 gas/solar turbines or open-cycle industrial units;
- 6 combined-cycle units exploit the heat emitted from a pair of gas turbines and use it to power a third turbine; these combined-cycle units operate at relatively high efficiency levels;
- 40 jet turbines were used primarily as backup because of high generating costs;
- 19 private companies with licenses for independent generation with total installed capacity of 353 MW (approximately 3.5% of the total generation);
- one wind station with installed capacity of 6 MW in the Golan Heights and 1 photovoltaic station with installed capacity of 5 MW in the Arava Region.

**Figure 1:**
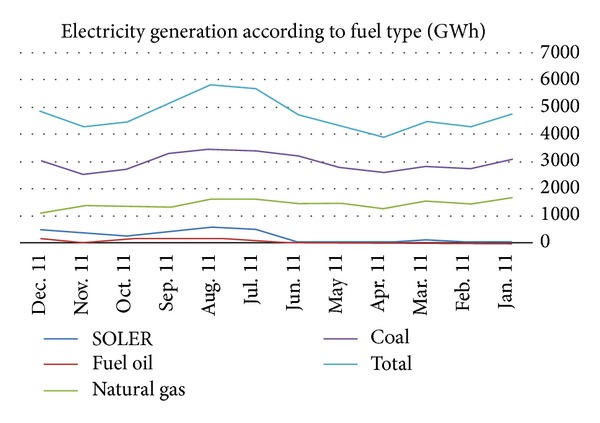
2011 generation according to fuel type.

In addition, there are several photovoltaic facilities of 200 MW installed capacity for self-consumption. Clearly, these systems are nondispatchable and require backup and techniques for reducing the undesirable phenomena resulting from the intermittency of electricity produced from these stations.  Table 1 summarizes the actual reaction times for each mentioned technology type.  Figure 1 presents the amount of generated electricity according to fuel type in 2011.

### 2.2. Consumption

**Figure 2:**
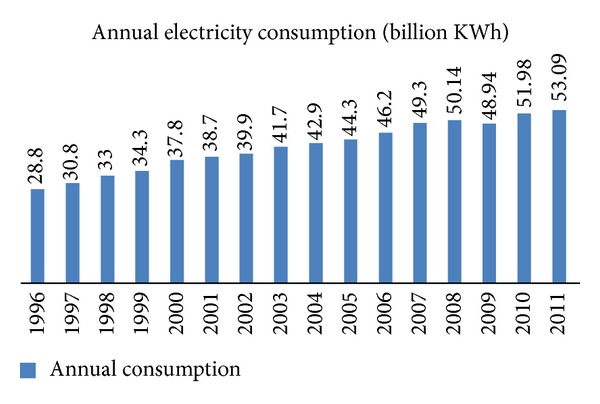
1996–2011 consumption.

The peak electricity demand of 11,920 MW occurred on July 19, 2012. For comparison purposes, the peak demand was 9,400 MW in 2006, 8,550 MW in 2004, and 7,900 MW in 2000; the consumption growth is evident. It is interesting to note that, during the 90s, the annual load growth rate was 7% per year, while, during the first decade of the 21st century, the annual load growth rate dropped to 4%. The annual consumption for the period of 1996–2011 is summarized in Figure 2.

### 2.3. Supply-Demand Balance

**Figure 3:**
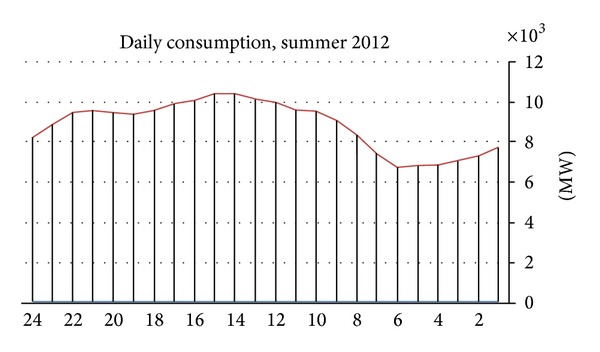
Hourly consumption, summer 2012.

Electricity demand is distributed over the course of the day according to economic activity. The base minimum consumption during the night is approximately 4,000 MW, reaching 8,000–9,000 MW during the day. Consumption rises during the morning hours (6:00–7:00) and returns to the nightly minimum at 23:00. The peak hours are between 11:00 and 17:00 during the summer and between 17:00 and 22:00 during the winter; see Figure 3.

As mentioned in the introduction, the Israeli electricity market operates as an “electricity island,” such that it is not connected to other networks that would make electricity imports/exports possible. Hence, the consumption distribution requires electricity generation to vary similarly over the course of the day. Moreover, an infrastructure for peak times that is not used during off-peak times must be established. Therefore, activities to straighten the consumption curve are required, reflected in TAOZ (the Hebrew abbreviation for time-differentiated tariff which is a tariff that varies according to the time of electricity consumption) rates. The consumption of large electricity consumers is measured according to the hours of the day and the consumer is obligated to pay the bill corresponding to this consumption. The day is divided into peak, shoulder, and off-peak hours. The tariff is intended first and foremost to reflect the costs of generation and to place those costs on the consuming party, while simultaneously making possible efficient and intelligent consumption decisions.

## 3. Investigation of the Government's Targets for Integrating Renewable Energy

In the Decision issued on July 17, 2011, the Government of Israel established a requirement to achieve a target whereby 10% of Israel's electricity consumption will come from renewable energy resources, in accordance with Decision no. 176 of January 12, 2009, and based on a policy document to integrate renewable resources issued by the Ministry of National Infrastructures in February 2010. Within this framework, the target was set as follows:

- 5% of the Israeli electricity market needs will be supplied by renewable resources by the year 2014. This target is expected to be achieved through installations totaling 1,550 MW.
- 10% of the Israeli electricity market needs will be supplied by renewable resources by the year 2020. This target is expected to be achieved through installations totaling 2,760 MW.

Table 2 details the breakdown of renewable energy by technology.

The Government targets are based on a comprehensive examination of all the natural resources in Israel, including the energy potential for wind and solar, since the potential for other renewables such as hydroelectric and biomass is insufficient. The forecast takes into account the developments in electricity demand in the Palestinian Authority that as of today receives electricity from Israel. In addition, the forecast accounts for the projected trend in energy efficiency. In any event, policy changes are possible as a result of economic and political changes.

## 4. Technical Issues Arising from Renewable Resources Intermittency in These Technologies and Possible Solutions

In an energywise isolated market, real-time forecast of renewable power stations production, including examining various backup options and providing tools to the system operator, is of enormous importance since the electricity production intermittency of nondispatchable wind and solar power stations may damage the level of reliability and survivability of the electric network. On the other hand, wider distribution and technology diversity can stabilize the overall process of renewable energy generation, and this can be a natural mitigator of the undesirable effects of renewable energy [9, 10].

Therefore, the current Capacity Credit substitution coefficient [4] between the installed capacity of fossil power stations and the installed capacity of solar and wind power stations [6] should be taken into consideration. The limited ability of the electricity grid to rely on renewable energy is evident from the facts that, for at least two of the past ten years, the peak demand occurred during evening hours in winter (when there is no solar-based generation) and the peak demand during the summer extended (albeit with a slight decrease) late into the evening hours. In addition, it is necessary to take into consideration the flexibility of the grid [5] using the Flexibility Factor (*ff*),

(1) $$\begin{array}{c} ff=1-\frac{L_{\min}}{L_{\max}}, \end{array}$$

which ranges, as calculated, between approximately 0.65 and 0.8 with *L*_min⁡_ and *L*_max⁡_ being the maximum and minimum loads in a given year. Grid flexibility determines, according to a specific model, the scope of renewable energy the grid can contain, taking into account its required operational (ramping) costs, without the need to disconnect and “dump” a portion of the electricity. Several possible solutions exist to the presented issues and are discussed next.

### 4.1. Improved Methods for Capacity Forecasting for Renewable Stations

Providing the system operator tools to build a model for renewable-station capacity forecasting based on information and weather data is as follows. For example, in case of wind generation stations, the instantaneous power output is given by [11, 12]

(2) $$\begin{array}{c} P_{g}=\eta \frac{\pi PD^{2}C_{p}v_{w}^{3}}{8RT}, \end{array}$$

where *η* is mechanical-to-electrical conversion efficiency, *P* is atmospheric pressure, *D* is blade diameter, *R* is ideal gas constant, *C*_*p*_ is turbine power coefficient, *T* is temperature, and *v*_*w*_ is instantaneous wind speed. For solar generation stations, the instantaneous power output is defined by [13, 14]

(3) $$\begin{array}{c} P_{g}=\eta \xi SR, \end{array}$$

where *η* is DC-to-AC conversion efficiency, *S* is the gross area of solar cell array, *R* is the solar irradiation incident upon the array, and *ξ* is the total efficiency of the photovoltaic array. Obviously that atmospheric conditions data is essential to utilize (2) and (3).

### 4.2. Incentivizing Flexible Stations

Establishment of arrangements incentivizing more flexible stations containing, for example, ultracapacitor installations, providing temporary storage ability on the DC side of the photovoltaic systems is done, minimizing the installations' intermittency and other dynamic effects on the grid [15, 16].

### 4.3. Development of Storage Technologies

At this stage, there is only one feasible technology allowing backup, namely, solar-thermal stations with molten salt heat storage. With rapid advances in technology, it is likely that in the near future the possibility of additional economically feasible electronic storage solution will be found, for example, storage solutions based on a combination of batteries and ultracapacitors [13]. This combination allows hybrid storage of energy in a highly efficient manner.

Solutions *B* and *C* require the storage types to be divided according to technologies and applications. Selection of a storage method depends on its application/implementation on the grid, the amount of renewable energy that is produced, and its stability in comparison/relation to the amount of fossil-fuel energy. These applications are largely determined according to energy discharge time. There are generally three regimes of storage applications, based on discharge time, as indicated in Table 3.

**Figure 4:**
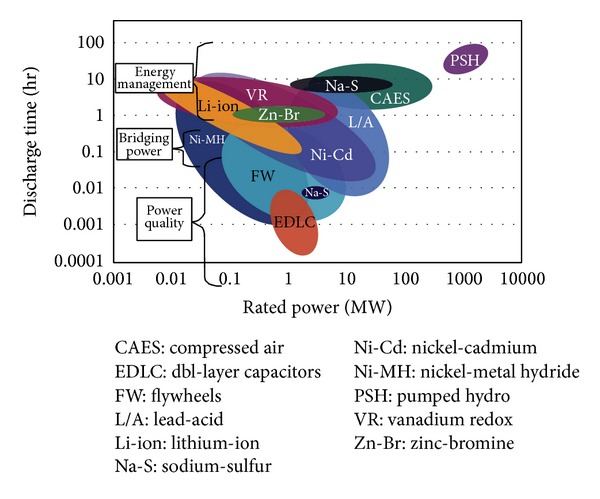
Ragone plot.

The Ragone plot [17], shown in Figure 4, provides various examples of technologies for the three types of applications and illustrates several technologies that can supply services over a variety of time frames.

## 5. Research Approach (Methodology)

**Figure 5:**
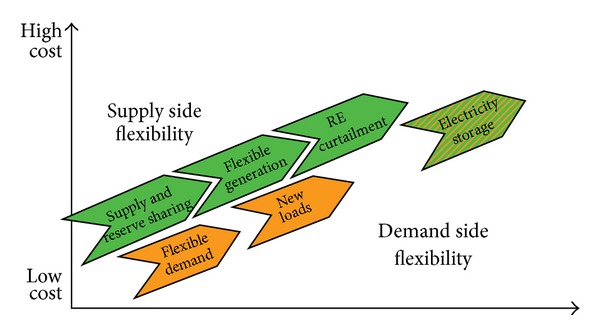
Different options for incorporating greater amount of renewable energy into the grid.

For purposes of calculating the fuel savings and the need for backup resulting from the increased use of renewable energy, Unit Commitment Optimal Dispatch software (UCOD) will be used. The idea is to determine the best way to respond to the varying electricity demand, which exhibits both daily and weekly cycles. The short-term optimization problem is how to schedule the activation of the stations in a way that it minimizes overall fuel costs or maximizes the overall profit, subject to a large number of system constraints, as shown in Figure 5.

The software is based on a proprietary multistage version of a mathematically rigorous optimization method, namely, the Mixed Integer Linear Programming (MILP), possessing the following advantages.

- The schedule produced by the software is always feasible; that is, it satisfies all the constraints modelled by the software, if the data are self-consistent. (If the data or constraints are not self-consistent, then a feasible solution does not exist; in such cases, the less important constraints are relaxed automatically by the software so that a usable schedule is always produced.)
- The schedules produced are better than those that could be found manually or by “priority-order” methods, leading to large savings in annual fuel costs and higher annual profits.
- A wide variety of constraints and plant types can be modelled, including complicated scheduling constraints, nonlinear cost curves, energy-limited plant, power-purchase agreements, and emission constraints.
- It is relatively easy and quick to introduce new constraints and features and to modify the software as circumstances change.
- The software is robust to changes in operating conditions and relative fuel costs, because no prior assumptions are made about the nature of the solution.

**Figure 6:**
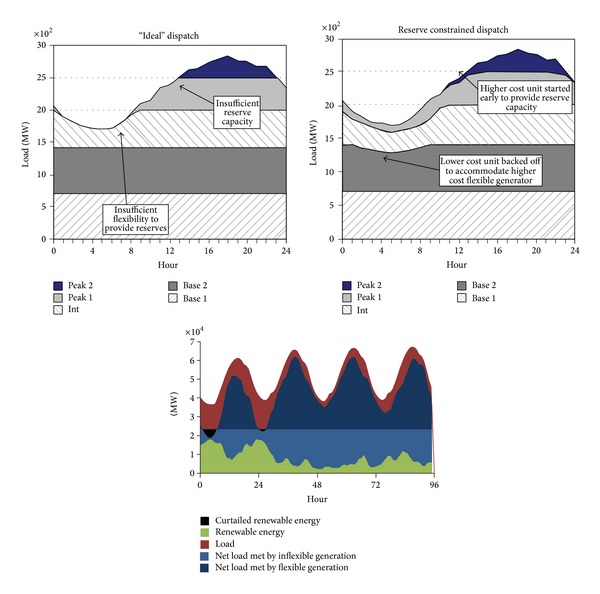
Example of UCOD based results.

As a result of using the proprietary multistage version of the MILP method, the software finds good feasible schedules much faster than if it were to use the standard MILP method. An example of software outputs is shown in Figure 6.

In order to calculate the amount of backup of renewable energy required on the generation and transmission systems, SAS (Statistical Analysis System) will be used to calculate the static coincidence factor for all the planned renewable-energy technologies combined, taking into consideration the following considerations.

- *Transmission capacity*. A transmission system which includes renewable-energy stations is more complex than one with conventional stations only, because of the low load factor which requires more backup.
- *Operational reserves*. If there is insufficient wind and sun, then the system operator must find alternatives in real time or to instruct the fossil-fuel stations to supply reserves; both of these options are likely to be costly.
- *Balance*. In order to maintain the stability of the system including voltage stabilization, wind and solar intermittency create additional requirements balancing resources in real time, such as pumped storage.
- *Storage*. Energy storage depends on its application on the grid, on the amount of generated renewable energy, and on its stability relative to fossil resources. These applications are largely determined according to discharge time. Generally, each application falls into one of three categories, based on discharge time.

To estimate the required backup level, there are a number of issues to be addressed, including the following.

1. Reconsidering the use of LOLP (Loss-of-Load Probability) as the measure that determines the maximum probability of blackout: if this measure is too conservative, the investments in handling the intermittency are less economically worthwhile.
2. Improvements in capacity forecasting for renewables stations: because of the large number of renewable stations and the experience gained over time, there are more tools available to the system operator to forecast the expected production levels from these stations. These tools include simulation forecasts of wind speed and sunlight, as well as neural-network-based software. In a number of models, it is possible to handle several distributed stations as one group and thus minimize errors. Each improvement in forecasting decreases the need for backup.
3. The encouragement of flexible stations, development of storage technology, and demand-side resources: in order to achieve the required backup levels, systems in Europe rely on connections with neighboring transmission systems and/or on running nonflexible stations when activating them creates excessive costs and less efficient operations.

## 6. Conclusion

The increasing use of renewable energy requires additional techniques and analyses of the economic and reliability impacts on the system in order to give the system operator tools to compensate for the intermittency of renewables in real time, while considering the actual reaction times of fossil generation units and pumped storage as well as their availability to back up the renewables.

In other research conducted in the area, it has been found that approximately 30% of the total installed capacity may be integrated into the electricity network without need for constructing a backup system, but this requires a change in operating procedures including the possibility of reducing the supply from renewable energy by 5% in real time. However, it is possible to state initially prior to publishing the results of the model runs to be described in this research that it is possible to integrate 20% of the installed capacity from renewables without any backup, albeit while considering correct distribution and use of techniques such as improving forecasting energy that can be generated by renewables and developing approaches for flexible consumption, as well as encouraging flexible generating stations.

It is clear that high penetration of renewable energy increases the need for all flexible options including storage, which also creates market opportunities for these technologies, both in terms of economic and technical competitiveness. At this stage, it is difficult to integrate a storage system into the market, not only because of the high cost but also because of the presence of innovative technologies mainly operating techniques such as improving the approaches to forecasting the supply from renewable stations and distributing stations among locations with different atmospheric conditions.

The challenge of simulating energy storage on the network, by setting forth mechanisms for smart consumption and encouraging flexible generation, is especially implementable in light of developments in communications and the smart grid, while it is clear to all that, in parallel with developing storage approaches, it is necessary to develop smart grids that will be able to provide a solution and tools for the system operator to overcome the undesirable phenomena stemming from the intermittency of renewable energy. Nevertheless, the value of energy storage is captured best with additional renewable energy generation and after a reduction in the use of conventional generation. Valuing the function of storage with renewable sources requires continued analysis, improved data, and new techniques in order to assess the activity of a smarter and more dynamic network in the future.

In the future work, the quantitative results of the study will be presented, determining the amount of backup required in order to maintain the reliability, survivability, and quality of electricity supply in a closed market such as the Israeli electricity system.

## Figures and Tables

**Table 1:** Reaction time of generating facilities.

Facility type	Installed capacity MW	Normal ramp rate MW/min	Accelerated ramp rate MW/min	Additional remarks
Gas/diesel turbine	280	0.7–1	1.5–3	Start-up time of 5 to 20 hours according to the unit's heat
Coal steam	360∗4	3	5	Warm start-up time of 8 hours and cold start-time of 72 hours
Coal steam	575∗4	5	10	Warm start-up time of 2 hours and cold start-time of 13 hours
Gas/diesel steam	214∗2	2-3	2-3	Start-up time of 5 to 20 hours according to the unit's temperature
Gas/diesel steam	228∗4	1	3-4	Start-up time of 3 to 20 hours according to the unit's temperature
Coal steam	575∗4	5	10	
Simple-cycle gas turbine	100∗2	10		Time from start-up to standing synchronization of 16 minutes
Combined-cycle gas turbine	336	10–20		Start-up time of 55 to 180 minutes, depending upon the facility's temperature
Simple-cycle gas turbine	110∗4	10		Synchronization time of 16 minutes
Combined-cycle gas turbine	340∗2	5–20		48 minutes to full load from warm start and 240 minutes from a cold start (i.e., not operating for at least 48 hours)
Simple-cycle gas turbine	148∗4	20		Synchronization time of 6 minutes
Jet turbines	40	Full load within 1–5 minutes		Synchronization time of 15 minutes
Combined-cycle gas turbine Model F	360–400	10	20	

**Table 2:** Renewable energy sources penetration by 2020.

Technology	Approved quota (MW)
Wind	830
Biogas and biomass	210
Solar thermal to the transmission grid	200
Photovoltaic to the transmission grid	260
Photovoltaic to the distribution grid	360
Photovoltaic up to 50 KW	200
Ashalim photovoltaic tenders to transmission grid	250
Additional quota for own consumption	110
Experimental/innovative technologies to the distribution grid	50

Total	2,470

**Table 3:** Features of desired energy storage.

Application	Desired feature	Required discharge time
Power quality	Transient stability, frequency regulation	Seconds to minutes
Bridging power	Contingency reserves, ramping	Minutes to ~1 hour
Energy management	Load leveling, firm capacity, *T*&*D* deferral	Hours
